# Novel Roles of Epoxyeicosanoids in Regulating Cardiac Mitochondria

**DOI:** 10.1371/journal.pone.0160380

**Published:** 2016-08-05

**Authors:** Haitham E. El-Sikhry, Nasser Alsaleh, Rambabu Dakarapu, John R. Falck, John M. Seubert

**Affiliations:** 1 Faculty of Pharmacy and Pharmaceutical Sciences, University of Alberta, Edmonton, AB, Canada; 2 Departments of Biochemistry and Pharmacology, University of Texas Southwestern Medical Center, Dallas, Texas, United States of America; 3 Department of Pharmacology, Faculty of Medicine and Dentistry, University of Alberta, Edmonton, AB, Canada; Hokkaido Daigaku, JAPAN

## Abstract

Maintenance of a healthy pool of mitochondria is important for the function and survival of terminally differentiated cells such as cardiomyocytes. Epoxyeicosatrienoic acids (EETs) are epoxy lipids derived from metabolism of arachidonic acid by cytochrome P450 epoxygenases. We have previously shown that EETs trigger a protective response limiting mitochondrial dysfunction and reducing cellular death. The aim of this study was to investigate whether EET-mediated effects influence mitochondrial quality in HL-1 cardiac cells during starvation. HL-1 cells were subjected to serum- and amino acid free conditions for 24h. We employed a dual-acting synthetic analog UA-8 (13-(3-propylureido)tridec-8-enoic acid), possessing both EET-mimetic and soluble epoxide hydrolase (sEH) inhibitory properties, or 14,15-EET as model EET molecules. We demonstrated that EET-mediated events significantly improved mitochondrial function as assessed by preservation of the ADP/ATP ratio and oxidative respiratory capacity. Starvation induced mitochondrial hyperfusion observed in control cells was attenuated by UA-8. However, EET-mediated events did not affect the expression of mitochondrial dynamic proteins Fis1, DRP-1 or Mfn2. Rather we observed increased levels of OPA-1 oligomers and increased mitochondrial cristae density, which correlated with the preserved mitochondrial function. Increased DNA binding activity of pCREB and Nrf1/2 and increased SIRT1 activity together with elevated mitochondrial proteins suggest EET-mediated events led to preserved mitobiogenesis. Thus, we provide new evidence for EET-mediated events that preserve a healthier pool of mitochondria in cardiac cells following starvation-induced stress.

## Introduction

Mitochondria provide the primary source of energy that fuels the contractile apparatus within the heart and have a key role in regulating cellular death pathways. These dynamic organelles respond to changes in cellular energy demands and stress levels [1–3]. As cardiomyocytes are post-mitotic cells, maintenance of a healthy pool of mitochondria depends upon a delicate balance between newly generated organelles and efficient turnover of irreversibly damaged ones [4, 5]. Mitochondrial quality control is maintained through precise coordination of a complex interplay between mitobiogenesis and selective degradation of through autophagic processes. Dysfunctional mitochondria disturb the energetic balance in the myocardium and can initiate cell death [6]. The removal of dysfunctional mitochondria is an important process to maintain a robust mitochondrial network within the cardiomyocyte [7, 8]. Indeed, compromised mitochondrial quality is linked to major cardiovascular pathologies such as heart failure and ischemic heart disease [9, 10]. Thus, optimum mitochondrial health is vital for cardiomyocyte performance and resistance to stress.

Mitochondria are a major site of interaction between energy metabolism and cell survival pathways, therefore, their response to cellular metabolic stress shapes the cellular fate [11]. During nutrient restriction and other forms of cellular stress, mitochondria fuse into elongated hyperfused networks, which is termed Stress Induced Mitochondrial Hyperfusion (SIMH) [12–14]. The transient hyperfused adaptation provides protection against apoptosis and spares mitochondria from autophagic degradation [15, 16]. However, sustained mitochondrial hyperfusion has been reported to induce inflammatory and apoptotic pathways [17, 18]. Importantly, the majority of reports concerning mitochondrial response to cellular stress have been conducted in non-cardiac cells.

An important regulator of mitochondrial dynamics and function is the Optic Atrophy 1 (OPA1). OPA1 is a large GTPase associated with the mitochondrial inner membrane playing key roles in regulating mitochondrial function and quality control by blocking the fusion of dysfunctional mitochondria segregating them for autophagic removal [19–22]. Recent studies demonstrate that OPA1 regulates mitochondrial reaction to cellular stress [23, 24]. This OPA1-dependent stress response adapts mitochondrial respiration and apoptotic resistance according to the metabolic demand and the cellular stress status. Despite the mounting evidence that OPA1 is a key regulator of mitochondrial function, very little is known about the role it plays in cardiovascular health.

Epoxyeicosatrienoic acids (EETs) are cytochrome P450-dependent epoxides of arachidonic acid that possess autocrine and paracrine signaling activity regulating a wide range of cellular functions [25, 26]. Several reports indicate a protective effect of EETs toward cardiac mitochondria [27–30]. We recently reported EETs enhance an autophagic response in cardiac cells promoting their survival during starvation [31]. However, it was unknown how EET-mediated signaling preserved mitochondrial tolerance to starvation. In the current study, we further investigate the role of EETs in regulating cardiac mitochondria and protecting cells during starvation stress. We report that EETs are actively involved in the regulation of mitochondrial quality providing novel insight into EET-mediated cardioprotective mechanisms.

## Methods

### Cell culture and treatment protocol

HL-1 cardiac cells were cultivated at 37°C in a humidified atmosphere of 5% CO_2_ and 95% air in Claycomb media (Sigma-Aldrich, Oakville, ON) supplemented with 10% fetal bovine serum (FBS, Batch#14E332, Sigma-Aldrich, Oakville, ON), glutamine (2mM) and norephinephrine (0.1mM) (cells were a kind gift from Dr. Claycomb, New Orleans, LA, USA) [31]. Starvation was modulated by incubating cells in serum- and amino acids-free buffer as described [32]. In this study, we utilized a novel EET-analogue, UA-8 (13-(3-propylureido)tridec-8-enoic acid, 1μM)) that possesses EET-mimetic and sEH inhibitory properties [33]. In order to block EET-mediated effects, we utilized the antagonist, 14,15-epoxyeicosa-5(Z)-enoic acid (14,15-EEZE, 10μM). Control experiments utilized 14,15-EET (1μM) as a model epoxyeicosanoid.

### Western blot assay and ELISA

HL-1 cells were treated as described above, washed with ice-cold phosphate buffer saline (PBS) and harvested using ice-cold lysis buffer (20 mM Tris-HCl, 50 mM NaCl, 50 mM NaF, 5 mM Na pyrophosphate, 0.25 M sucrose, 1 mM DTT, 1% triton-X100 and protease/phosphatase inhibitors). Cell lysates were incubated on ice for 10 min, then centrifuged at 13,000g for 15 min (4°C). The Bradford assay was used to measure total protein content in supernatants. 20 μg of protein was resolved in 10% SDS-polyacrylamide gel and then transferred electrophoretically to polyvinylidene fluoride membranes, which were then blocked with TBS-T buffer (0.15 M NaCl, 3 mM KCl, 25 mM tris hydroxymethyl methylamine and 0.1% tween-25, pH 7.4) with 5% skim milk for 3 h at room temperature. Membranes were washed three times with TBS-T buffer (with 15-min intervals) and then incubated with either mouse monoclonal antibody against cytochrome c oxidase subunit 4 (Cell Signaling Tech., Inc., Whitby, ON, cat#11967), rabbit polyclonal antibody against succinate dehydrogenase subunit A (Cell Signaling Tech., Inc., Whitby, ON, cat#5839), mouse monoclonal antibody against prohibitin (Fitzgerald #10R-P140A), rabbit monoclonal antibody against DRP1 (Cell Signaling Tech., Inc., Whitby, ON, cat#8570), rabbit polyclonal antibody against phospho-DRP1 (Ser637) (Cell Signaling Tech., Inc., Whitby, ON, cat#4867), rabbit polyclonal antibody against Fis1 (Novus Biologicals Canada, ULC, Oakville, ON, cat#IMG-5113A), rabbit monoclonal antibody against Mfn2 (Cell Signaling Tech., Inc., Whitby, ON, cat#9482), mouse monoclonal antibody against OPA1 (BD Biosciences, Mississauga, ON, cat#612606) or rabbit polyclonal antibody against α-tubulin (Abcam., Cambridge, UK, cat#4074) overnight at 4°C. Membranes were rinsed as described above and incubated with horseradish-peroxidase linked anti-rabbit or anti-mouse IgG secondary antibody (Invitrogen, CAN) for 2 h at room temperature followed by washing as described above. Chemiluminscent solution was used to detect the antibody-protein complexes. Relative band intensity to control was measured using Image J software (NIH, USA). An In-Cell ELISA kit (Abcam., Cambridge, UK cat#ab110217) was used to assess protein levels of succinate dehydrogenase (SDH-A), a subunit of complex II (nDNA-encoded protein) and cytochrome c oxidase subunit 1 (COX-1), a subunit of complex IV (mtDNA-encoded).

### Aconitase catalytic activity

Mitochondrial aconitase enzymatic activities were measured spectrophotometrically utilizing MitoSciences kit (Abcam, Cambridge, UK, cat#ab109712) as previously described [34]. Briefly, mitochondria were isolated from HL-1 cells following starvation protocol and approximately 50μg of mitochondrial preparation were placed in each microplate well. Equal amounts of the substrate isocitrate were added to all wells and the absorbance at 240nm was recorded for 30 minutes. The catalytic activity was measured by the rate of formation of cis-aconitate as detected by the increase in absorbance.

### SIRT1 enzymatic activity

SIRT1 deacetylation activity was measured by a chemo-luminescence method using a commercially available SIRT-Glo kit (Promega Corp., Madison, WI, cat#6450). Whole cell lysates (with and without the inhibitor Trichostatin A (10mM)) were incubated with an acetylated, luminogenic peptide substrate that can be deacetylated by SIRT activities.

### Mitochondrial respiration and cellular energy levels

Mitochondrial oxygen consumption was measured in permeabilized cells as previously described [35]. Precisely 250μg protein of the cell suspension was loaded and incubated for 10 min in a chamber connected to the Oxygraph, which contained 2 ml of the respiration medium added. Respiration medium (EGTA 0.5 mM, MgCl_2_.6H_2_O 3 mM, taurine 20 mM, KH_2_PO_4_ 10 mM, HEPES 20 mM, BSA 0.1%, potassium-lactobionate 60 mM, mannitol 110 mM, dithiothreitol 0.3mM, pH 7.1, adjusted with 5 N KOH) also contained 25μg/mL of saponin to ensure an effective permeabilization occurring within 10 min 30°C. Complex I substrates glutamate (10mM) and malate (5mM) were then added and oxygen consumption was recorded in the absence of ADP (State 4 respiration). ADP-stimulated respiration rate was then determined with the addition of 1mM ADP (State 3 respiration). Respiration rate was represented as nmole O_2_ consumed per mg protein per minute. The ratio between basal and ADP stimulated respiration rates was expressed as respiratory control ratio (RCR).

To measure cellular energy levels, we used a bioluminescence-based kit (Sigma–Aldrich, Oakville, ON, CAN) to assay the ratio between ATP and ADP in HL-1 cells as described previously [36]. Briefly, cells were cultured on a 96-well plate then treated accordingly to the starvation protocol. The culture medium was then removed and the ATP assay reagent was added containing D-luciferin substrate and the luciferase enzyme. ATP immediately reacts with the substrate producing light that was measured with a luminometer. ADP assay reagent was then added to enzymatically convert ADP to ATP which then reacted with the substrate and produced additional light reflecting the ADP content.

### DNA-binding activity of pCREB, Nrf1 and Nrf2

The DNA binding activity of transcription factors CREB, Nrf1 and Nrf2 were measured by their ability to bind to their corresponding target DNA sequences. Specified ELISA kits were used for pCREB-Ser133 (Cayman Chemicals, Ann Arbor, MI, USA), Nrf1 (Assay BioTech, Sunnyvale, CA, USA) and Nrf2 (Active Motif, Carlsbad, CA, USA). Briefly, following treatment protocol, cells were lysed and the nuclear fraction was extracted according to the assay protocols. Equal protein amounts were incubated on assay plates coated with the corresponding DNA sequence specific to the transcription factor. Only active transcription factors bind to the DNA-coated plates allowing their immunodetection using the provided antibodies. The substrate TMB (3, 3’, 5, 5’-Tetramethylbenzidine) was added as a substrate to the HRP-linked secondary antibodies producing a blue color. The reaction was then stopped by sulfuric acid and absorbance was measured at 450nm indicating the bound transcription factors.

### Mitochondrial morphometric analysis

**Fig 1:**
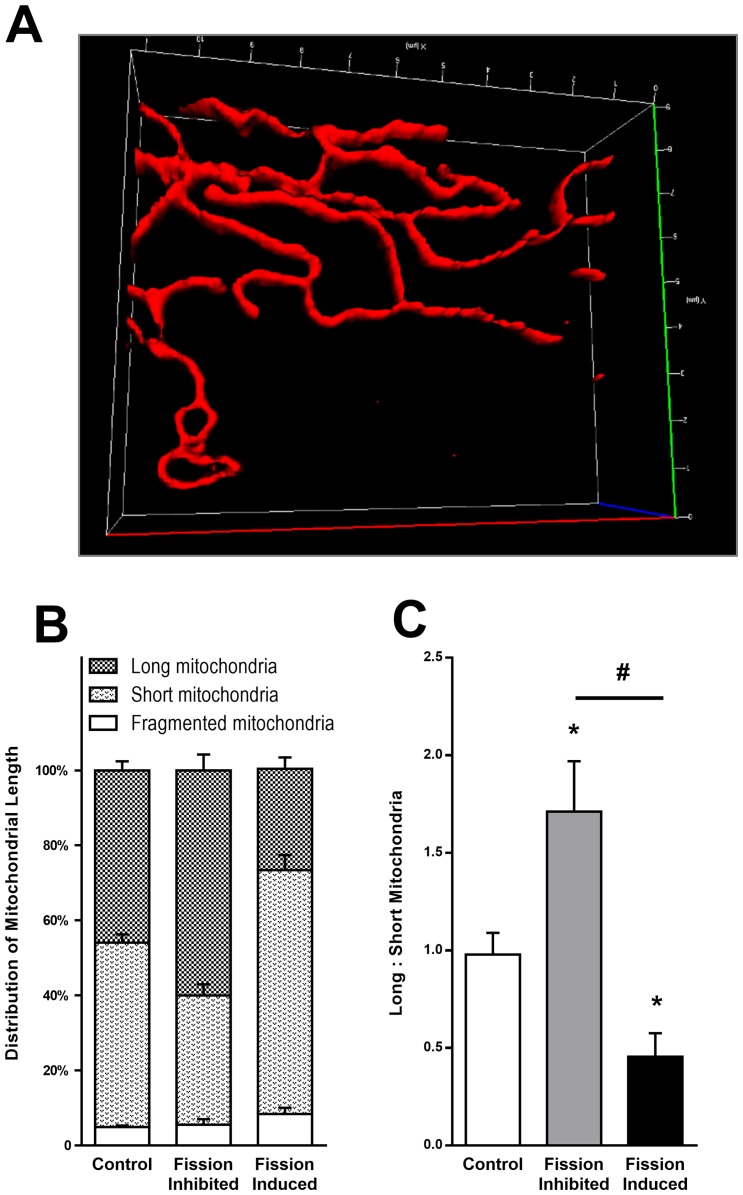
Morphometric analysis of mitochondrial dynamics. **A.** 3D images of mitochondria were analyzed morphometrically to identify mitochondrial structures and to numerically report their length and size. Mitochondria were stained with MitoTracker Red (100nM) and Z-stacks images were captured with Zeiss AxioObserver Epifluorescence microscope. Image processing was conducted by Zeiss ZEN 12 and Bitplane Imaris software as detailed in the methods section. **B.** Distribution of mitochondria according to their length into long (>5μm), short (<5μm) and fragmented (<0.5μM) mitochondria. To verify the morphometric method in our cells, DRP1-dependent fission was inhibited by forskolin (20μM) and cyclosporine A (10μM) or activated by H89 (1μM) and iononmycin (5μM). **C.** The ratio of long mitochondria to short and fragmented mitochondria provides a single descriptor to mitochondrial length allowing simple statistical analysis between treatment groups. Values represent mean+SEM; *N* = 3 independent experiments, *, *p<*0.05 in comparison to control.

We employed a morphometric analysis approach to track filament-like structures and characterize 3-dimensional morphology of individual mitochondria and networks [37]. HL-1 cells were grown on glass-bottom 35-mm dishes or 6-well microplates suitable for fluorescent microscopy (MatTek) and treated as outlined above. Next, mitochondria were stained with MitoTracker Red (100nM) or TMRE (100nM) and placed in a microincubator installed on the objective stage of a Zeiss AxioObserver epifluorescence microscope. A series of Z-stack images were acquired at optimal z intervals with a Plan-Apochromat 63x/1.4 oil immersion objective lens. A minimum of 5 random spots, each containing an average of 10 cells, were imaged per treatment group. Experiments were repeated at least 3 independent times and images contained a minimum of 100 cells per treatment group and files were renamed for blind morphometric analysis. Images were first de-convoluted and 3D images of the mitochondrial network were reconstructed using Bitplane Imaris software. The FilamentTracer module was used to track mitochondrial voxels in the 3D space to identify individual mitochondrial bodies and characterize their morphology. The Threshold (loops) algorithm was used to detect mitochondrial fluorescence signal and the approximate diameter was set to ignore signals less than 0.2μm improving signal to noise ratio. Mitochondria were categorized according to their length into three categories; fragmented mitochondria (0.2–0.5μm), short-to-medium length mitochondria (0.5–5μm) and long-branched mitochondria (>5μm), as reported previously [37]. To better characterize the distribution of mitochondria between the categories, the volume of mitochondria were compared to overall total mitochondrial of the population. The ratio between the volume of all long mitochondria to the volume of short and fragmented mitochondria combined was used to compare mitochondrial populations (Fig 1A and 1B). Validation was performed by treating HL-1 cells with positive or negative controls for the DRP1-dependent mitochondrial fission (Fig 1C). To activate mitochondrial fission, cells were treated with the PKA inhibitor H89 (1μM) and the calcium ionophore ionomycin (5μM). To inhibit fission and promote mitochondrial elongation, cells were treated with the adenyl cyclase activator forskolin (20μM) and the calcineurin inhibitor cyclosporin A (10μM).

### Assessment of OPA1 oligomers

OPA1 oligomers were stabilized with protein crosslinking and assessed with immunoblotting as described [19, 38]. Briefly, following treatment protocol, cells were washed twice with washing buffer (in mM; 120 KCl, 5 KH_2_PO_4_, 10 HEPES, 1 MgSO_4_, and 2 EGTA, pH 7.4), then incubated for 30min at 37°C in the reaction buffer (50 μg/mL digitonin and 20 mM EDC in washing buffer). 1-Ethyl-3-[3-dimethylaminopropyl] carbodiimide hydrochloride (EDC) was used as a cross linker for zero-length, carboxyl-to-amine conjugation to forms stable covalent bonds between OPA1 molecules in the oligomer enabling their detection with SDS-PAGE analysis [19, 38]. Lysis buffer was then added containing 50mM dithiothreitol (DTT) to quench the crosslinking reaction [19]. Cell lysates were gathered for protein assay and SDS-PAGE using 7% gels.

### Assessing cristae density

HL-1 cells were grown and treated on glass-bottom dishes (MatTek). Cells were prepared for transmission electron microscopy immediately after experiment as previously described [31]. Briefly, the cell monolayer was fixed with Karnovsky (2% glutharaldehyde and 2% paraformaldehyde), post-fixed in 1% osmium tetroxide and stained in 2% unanyl acetate prior to dehydration and blocking. Electron micrographs were acquired with the Philips 410 electron microscope at 17000x. The experiment was repeated 3 times and electomicrographs were renamed for blind analysis. The density of cristae folds in the mitochondria was assessed by counting number of cristae membranes per perpendicular unit of distance (μm).

### Statistical analysis

Data are presented as mean ± SEM. Statistical analysis was based on one-way ANOVA with a Tukey’s post hoc test; P<0.05 was considered statistically significant. Raw data obtained for the current study can be located in S1 File.

## Results

### EET-mediated effects toward mitochondrial and nuclear encoded proteins following starvation

**Fig 2:**
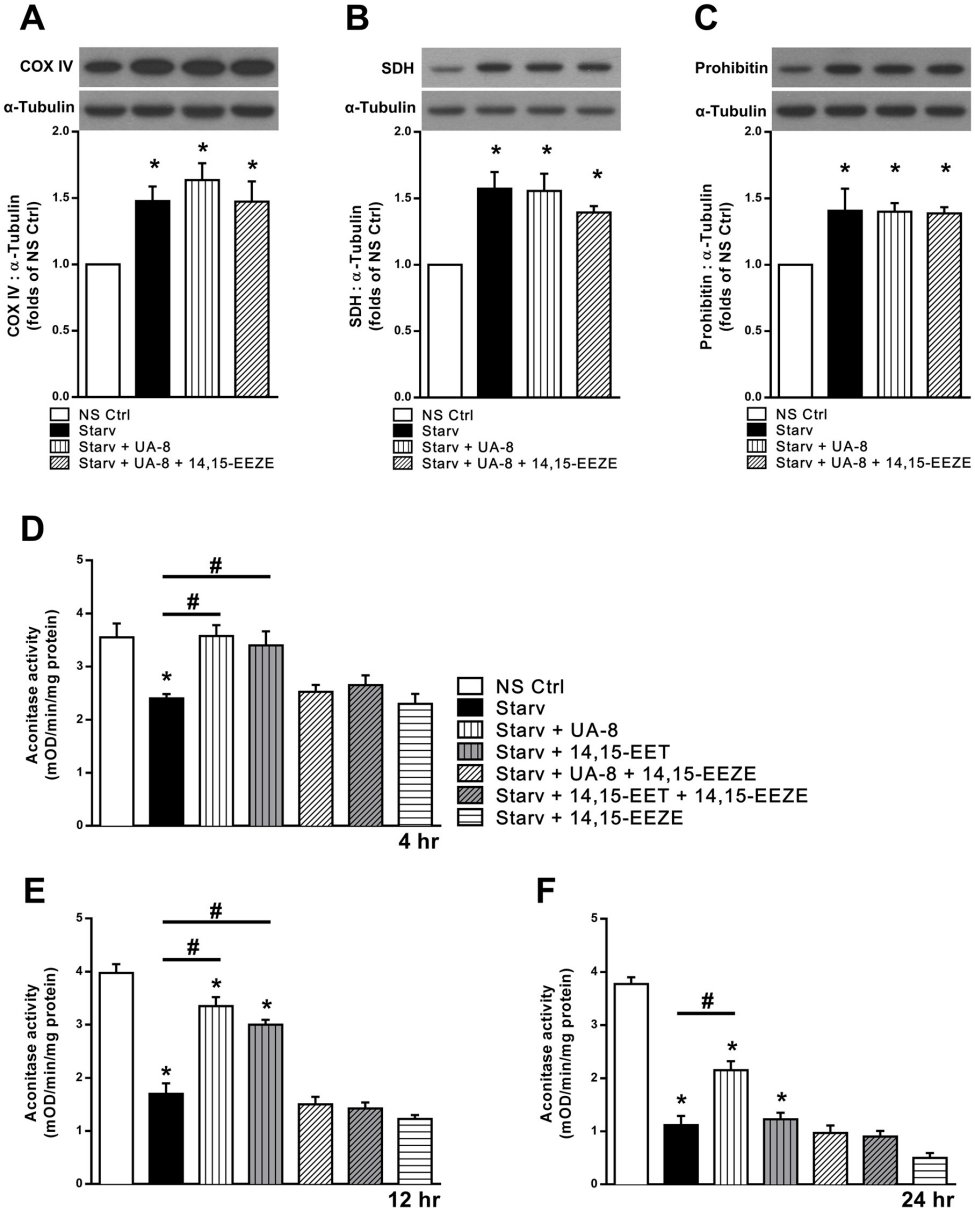
Mitochondrial enzyme expression and function following 24h starvation. Cardiac HL-1 cells were incubated either under normal conditions (NS Ctrl) or with an amino acid-free and serum-free starvation buffer (Starv) for 24h with or without UA-8 (1μM) and 14,15-EEZE (10μM). Protein expression in cells subjected to 24h starvation (**A**) cytochrome c oxidase subunit IV (COX-IV); (**B**), succinate dehydrogenase subunit A (SDH-A); and, (**C**) prohibition as demonstrated in representative immunoblots and quantified in corresponding histograms. (**D**) Protein expression of cytochrome c oxidase subunit I (COX-I) in cells subjected to 6h or 24h of starvation. (**E**) Aconitase enzymatic activity was measured in HL-1 cells after 6h or 24h of starvation. Starvation decreased activity of the mitochondrial enzyme while both 14,15-EET (1μM) and UA-8 (1μM) preserved activity. Co-treatment with the putative antagonist 14,15-EEZE (10μM) abolished the observed effect of 14,15-EET and UA-8. Values represent mean+SEM; *N* = 3 independent experiments, *, *p<*0.05 in comparison to non-starvation control, and, #, *p<*0.05 between indicated groups.

We first examined the expression level of nuclear encoded mitochondrial proteins, which tended to elevate levels of cytochrome c oxidase subunit 4 (COX-IV), succinate dehydrogenase A (SDH-A) and prohibitin (Fig 2A–2C) following 24h of starvation. The same effect was observed among all starved groups treated with UA-8 and/or 14,15-EEZE. These data are consistent with our previous results in neonatal cardiomyocytes [31]. In contrast, assessment of the mitochondrial encoded cytochrome c oxidase subunit 1 (COX-I) demonstrated an initial increase in expression at 6h, which was enhanced with both UA-8 and 14,15-EET (Fig 2D). However, following 24 h starvation COX-I expression was significantly down regulated (Fig 2D). Both UA-8 and 14,15-EET limited the deleterious effect of starvation thereby preserving expression levels relative to controls.

In the current report, we assessed aconitase activity, an important tricarboxylic acid cycle enzyme, in the presence of either UA-8 or 14,15-EET in HL-1 cells after 6 and 24h of starvation (Fig 2E). Due to its redox sensitivity, aconitase activity is commonly utilized to indicate mitochondrial oxidative stress [39, 40]. As well, evidence indicates aconitase assists in mtDNA maintenance and protection [41]. We observed a significant decrease in aconitase activity following 6h of starvation, whereas UA-8 and 14,15-EET treated cells had preserved activity. UA-8 treated cells continued to show higher aconitase activity compared to starved groups following 24h of starvation. Importantly, addition of the inhibitor 14,15-EEZE abolished the protective effect of UA-8 and 14,15-EET.

**Fig 3:**
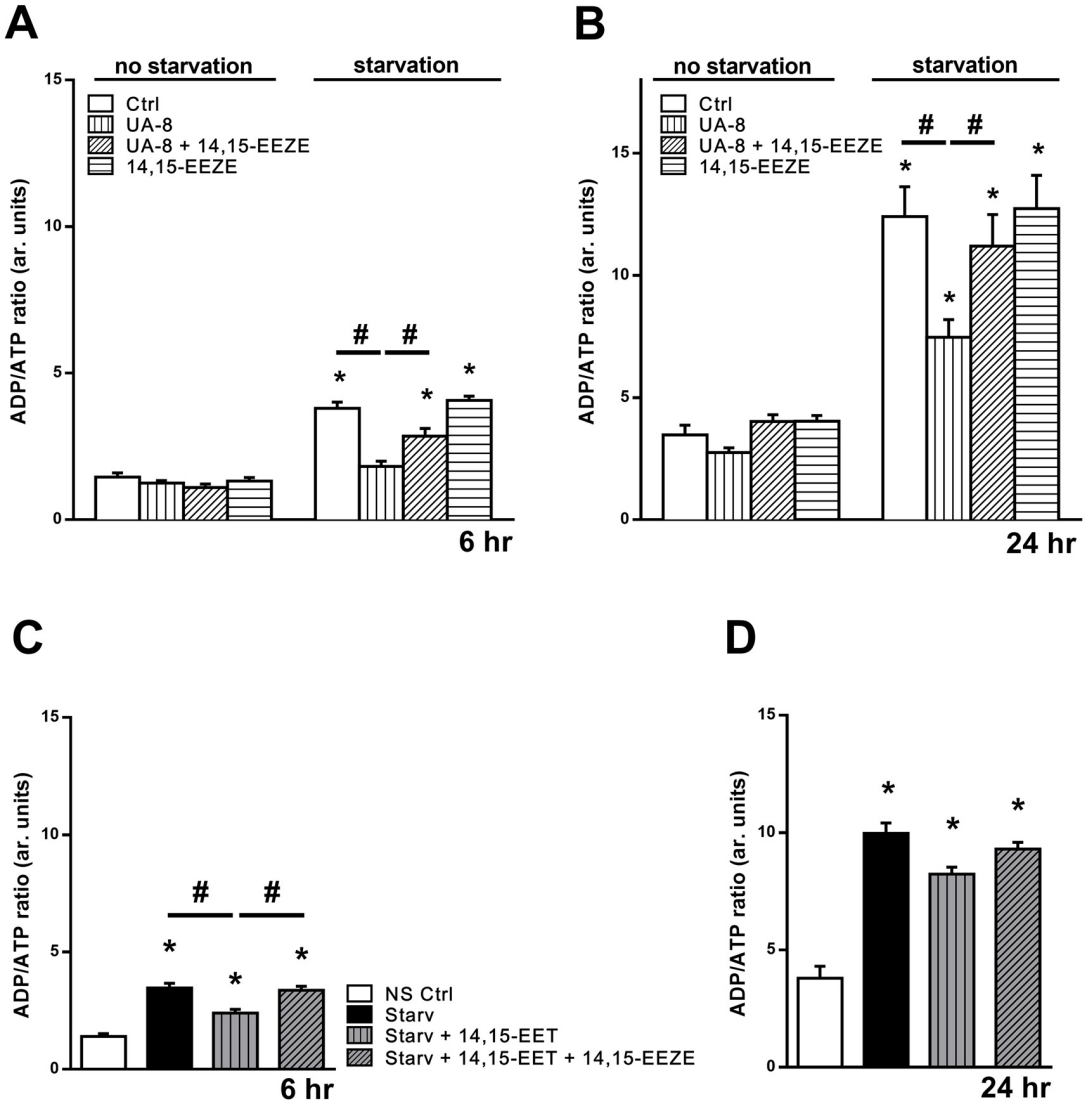
Preserved cellular energy levels in UA-8 and EET-treated cells. Intracellular levels of ATP and ADP were measured in HL1 cardiac cells by chemiluminescences and normalized μg protein. HL-1 cells were incubated in starvation buffer (Starv) with or without UA-8 (1μM) and/or 14,15-EEZE (10μM) for (**A**) 6h and (**B**) 24h. HL-1 cells were incubated in starvation buffer (Starv) with or without 14,15-EET (1μM) and/or 14,15-EEZE (10μM) for (**C**) 6h and (**C**) 24h. Values are expressed as ADP/ATP ratio reflecting the cellular energy demand. Values represent mean+SEM; *N* = 3 independent experiments, *, *p<*0.05 in comparison to non-starvation control, and, #, *p<*0.05 between indicated groups.

The coupling of oxygen consumption with generation of ATP, oxidative phosphorylation, is the most essential component of mitochondrial function. The ratio between basal mitochondrial oxygen consumption and ADP-stimulated states indicates the respiratory ratio control (RCR), which reflects mitochondrial bioenergetic efficiency. We measured the effect of starvation on mitochondrial respiration in permeabilized HL-1 cells. Starvation caused a significant decline in RCR, reflecting a collapse in mitochondrial function (Table 1). Importantly, cells treated with UA-8 showed a marked preservation in mitochondrial respiration, which was attenuated by 14,15-EEZE. Consistent with starvation-induced mitochondrial dysfunction, the cellular ratio of ADP/ATP significantly increased over the 24h time course (Fig 3). Treatment with UA-8 (Fig 3A and 3B) or 14,15-EET (Fig 3C and 3D) resulted in a significant reduction in the ADP/ATP ratio indicating preserved mitochondrial function.

**Table 1:** Mitochondrial respiration. The rate of oxygen consumption in different states of mitochondrial respiration. Cardiac HL-1 cells were incubated either under normal conditions or with an amino acid-free and serum-free starvation buffer for 24h with or without UA-8 (1μM) and 14,15-EEZE (10μM). Values represent mean ± SEM, N≥3, *, *p<*0.05 in comparison to non-starvation control, #, *p<*0.05 in comparison to starvation control.

		O_2_ Consumption (nmol O_2_/min/mg protein)		
		State 4	State 3	RCR
Non Starvation	Control	5.7 ± 1.0	25.2 ± 2.2	4.5 ± 0.3
	UA8	5.5 ± 0.9	32.2 ± 5.6	5.9 ± 0.3
	UA8+14,15-EEZE	6.2 ± 1.2	18.7 ± 6.2	3.4 ± 1.4
	14,15-EEZE	6.6 ± 1.6	18.7 ± 5.4	3.5 ± 1.4
24h Starvation	Control	3.7 ± 0.9	4.4 ± 0.7*	1.3 ± 0.2*
	UA8	4.1 ± 0.5	19.8 ± 2.4^**#**^	4.8 ± 0.2^**#**^
	UA8+14,15-EEZE	5.2 ± 1.6	5.0 ± 1.3*	1.0 ± 0.0*
	14,15-EEZE	2.9 ± 1.4	2.8 ± 1.4*	0.9 ± 0.1*

### UA-8 limits starvation induced mitochondrial hyperfusion

**Fig 4:**
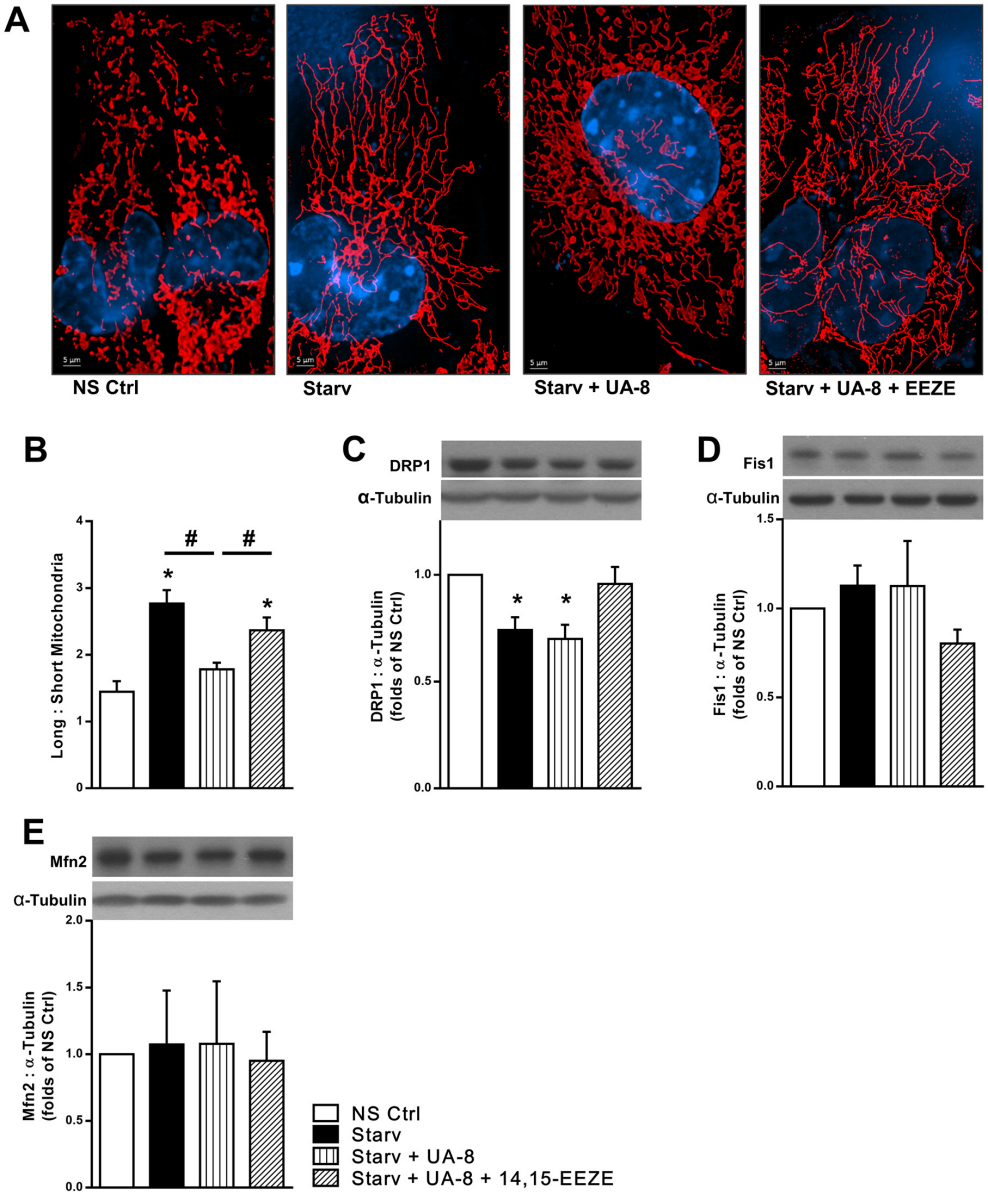
UA-8 inhibited starvation-induced mitochondrial hyperfusion. HL-1 cardiac cells were treated under normal cell culture conditions (NS Ctrl) or following 24h starvation (Starv) with or without UA-8 (1μM) and/or 14,15-EEZE (10μM). (**A**) Representative images of mitochondrial morphology demonstrating starvation induced mitochondrial hyperfusion that was inhibited by UA-8. (**B**) The ratio of long:short mitochondria reflecting the starvation-induced hyperfusion and the inhibitory effect of UA-8 (>25 cells per treatment group were assessed in each experiment). (**C**) Relative expression of the mitochondrial fission protein DRP1. (**D**) Relative expression of the mitochondrial fission protein Fis1. (**E**) Relative expression of the mitochondrial fusion protein Mfn2. Values represent mean+SEM; *N* = 3 independent experiments, *, *p<*0.05 in comparison to non-starvation control, and, #, *p<*0.05 between indicated groups.

Our results show a starvation-induced decline in mitochondrial function with limited loss in content. However, EET-mediated events led to better function suggesting a preserved pool of mitochondrial quality. It has been shown that starvation stress inhibits mitochondrial fission in non-cardiac cells leading to mitochondrial elongation and formation of hyperfused mitochondrial networks [15, 42]. As mitochondrial fission is required for proper mitochondrial quality control, we assessed mitochondrial dynamics in cardiac HL-1 cells following starvation. We optimized an automated morphometric analysis to define alterations in mitochondrial dynamic balance. Mitochondrial morphology of starved HL-1 cells confirmed the starvation-induced hyperfusion, which was sustained for 24 hours (Fig 4A). Mitochondrial particles and short tubes of non-starved cells turned into branched interconnected networks composing the majority of the cell’s mitochondrial volume. Interestingly, UA-8 treated cells showed significantly less mitochondrial hyperfusion, while co-treatment with 14,15-EEZE abolished the UA-8 effect (Fig 4A). Blind morphometric analysis of microscopical 3D images from three independent starvation experiments confirmed that UA-8 inhibited the starvation-induced mitochondrial hyperfusion (Fig 4B).

Fusion and fission processes are regulated by a group of proteins including DRP1, Fis1 and Mfn2. Immunoblot analysis of the relative levels of these proteins was performed following 24 hours of starvation. We found a significant reduction in mitochondrial pro-fission protein DRP1 in all starved groups (Fig 4C), which correlated with the observed hyperfusion in controls but not UA-8 treated. Fis1 and Mfn2 did not show a consistent response to either starvation or UA-8 (Fig 4E and 4F).

### UA-8 limits starvation-associated effects toward OPA1 oligomers and mitochondrial cristae density

**Fig 5:**
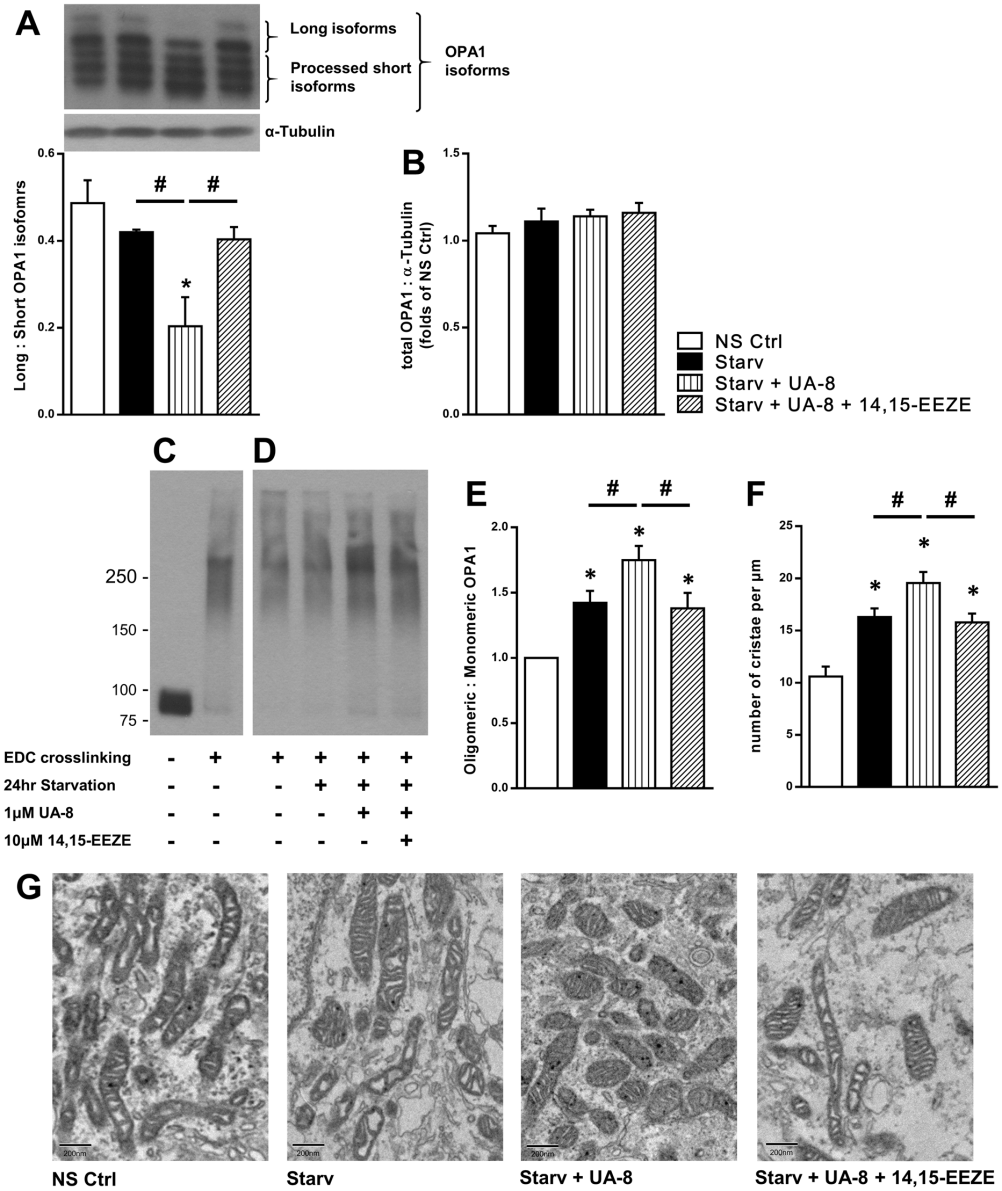
Effect of starvation and UA-8 treatment on OPA1 and cristae density. HL-1 cardiac cells were treated under normal cell culture conditions (NS Ctrl) or following 24h starvation (Starv) with or without UA-8 (1μM) and/or 14,15-EEZE (10μM). (**A**) UA-8 treated cells had lower levels of L-OPA1 isoforms and higher levels of S-OPA1 isoforms, while 14,15-EEZE inhibited this effect. (**B**) Histogram representing the quantification of total OPA1 expression. (**C**) Crosslinking OPA1 molecules with EDC (20mM) stabilized the oligomers and allowed their detection with SDS-PAGE analysis. (**D**) OPA1 oligomers as detected in HL-1 cells following 24h starvation with the indicated treatments. (**E**) Histogram representing the quantification of the ratio of oligomeric to monomeric OPA1. (**F)** Cristae density, presented by number of cristae per mitochondrial length, showing similar pattern to OPA1 oligomers. (**G**) Representative electromicrograph images depicting changes in cristae width and number upon starvation and UA8 treatment. Values represent mean+SEM; *N* = 3 independent experiments, *, *p<*0.05 in comparison to non-starvation control, and, #, *p<*0.05 between indicated groups.

OPA1 is a dynamin-like protein found in the inner mitochondrial membrane that regulates both mitochondrial fusion and cristae structure. Following 24h starvation, UA-8 treatment results in a decreased ratio of long isoform of OPA1 (L-OPA1) to short isoforms (S-OPA1, Fig 5A), while the total expression of all OPA1 isoforms did not significantly change (Fig 5B). Since S-OPA1 is the product of proteolytic processing of L-OPA1[43], these data indicate a stimulating effect of UA-8 on the processing of L-OPA1 to S-OPA1 during starvation. Furthermore, co-treatment with 14,15-EEZE attenuated the processing effect of UA-8 treatment toward OPA1 isoforms. L-OPA1 is required for mitochondrial hyperfusion [12]. HL-1 cells treated with UA-8 had reduced levels of L-OPA1 (Fig 5A), which correlated with the lower amount of hyperfused mitochondria observed during starvation (Fig 3A and 3B). In addition to a role in mitochondrial fusion, OPA1 molecules assemble into oligomers necessary for cristae organization [44]. Following the results of UA-8 on OPA1 isomers, we then looked at its effect on OPA1 oligomers. We assessed the effects of starvation and UA-8 treatment toward UA-8 oligomer levels. Stabilizing OPA1 oligomers with crosslinking (Fig 5D) allowed us to detect increased levels of OPA1 oligomers following starvation and UA-8 treatment. The higher level of oligomers following UA-8 was attenuated by co-treatment with 14,15-EEZE (Fig 5E).

Mitochondrial ultrastructure was assessed using electron microscopy in non-starved and starved cells following treatment. Mitochondria in non-starved cells had loose cristae arrangement with low number per mitochondrion, which was increased following 24h starvation (Fig 5F and 5G). Mitochondria in UA-8 treated cells had compact cristae structure using most of the space inside the mitochondrion, which was reduced by co-treatment with 14,15-EEZE (Fig 5F and 5G). OPA1 oligomers are important to cristae organization, which ultimately impacts mitochondrial respiratory efficiency [45]. These data suggest that EET-mediated signaling can regulate OPA1 oligomer levels and cristae density thus contributing to the preserved mitochondrial function in HL-1 cardiac cells.

### EETs Enhanced and Preserved Nrf1, Nrf2 and pCREB DNA Binding Activities

**Fig 6:**
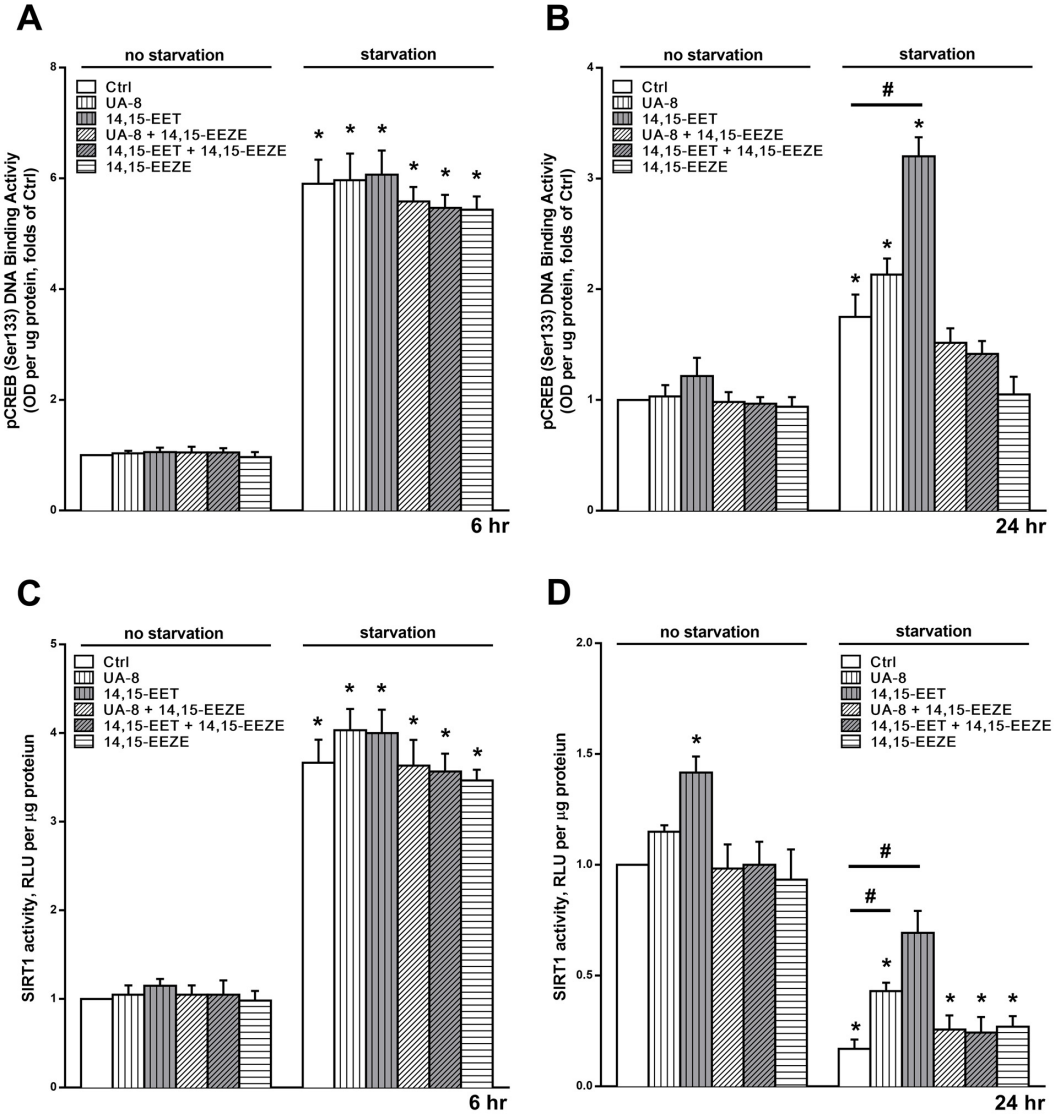
EETs induce mitobiogenesis in HL-1 cardiac cells. HL-1 cardiac cells were treated under normal cell culture conditions (NS Ctrl) or following 24h starvation (Starv) with or without UA-8 (1μM), 14,15-EET(1μM) and/or 14,15-EEZE (10μM). DNA-binding activity of the pCREB (Ser133) in HL-1 cells following (**A**) 6h and (**B**) 24h. The catalytic activity of SIRT1 was measured in whole-cell lysates by bioluminescent assay in the presence of trichostatin A (1μM) in HL-1 cells following (**C**) 6h and (**D**) 24h. EET-treated cells had significantly preserved pCREB and SIRT1 activities following 24h starvation. Values represent mean+SEM; *N* = 3 independent experiments, *, *p<*0.05 in comparison to non-starvation control, and, #, *p<*0.05 between indicated groups.

An important element of the mitochondrial quality is the biogenesis of mitochondrial proteins. EETs are recognized agonists for peroxisome proliferator-activated receptors (PPARs) [36, 46], which play a significant role in promoting expression of several mitochondrial proteins [47, 48] Recently, Wang et al. reported that 14,15-EET induced mitochondrial biogenesis in cortical neuronal cells via activation of cAMP-response element binding protein (CREB), which in turn activated the peroxisome proliferator-activated receptor gamma-coactivator 1 (PGC1) [49]. In order to investigate the involvement of mitochondrial biogenesis in the protective effect of EETs in HL-1 cardiac cells, we assessed several regulators of mitochondrial biogenesis following 6 and 24 hours of starvation. We measured DNA binding activity of pCREB (Ser133), an upstream activator of PGC1. Starvation induced a marked 6-fold activation of pCREB during the first few hours (Fig 6A), which subsided by 24h (Fig 6B). However, both 14,15-EET and UA-8 preserved pCREB activity (Fig 6B), an effect that was completely abolished with 14,15-EEZE co-treatment. Of note, pCREB in non-starved cells was not as sensitive to 14,15-EET or UA-8.

Next, we assessed the catalytic activity of the nuclear deacetylase SIRT1, which can deacetylate PGC1 resulting in activation of mitochondrial biogenesis [50]. We observed a time-dependent SIRT1 response to starvation-induced stress, with activation at 6h (Fig 6C) followed by a drop below non-starvation levels at 24h (Fig 6D). Interestingly, 14,15-EET and UA-8 preserved SIRT1 activity at the 24h point, which was attenuated by co-treatment with 14,15-EEZE (Fig 6D). We observed a moderate activation of SIRT1 activity in non-starved cells treated with 14,15-EET.

**Fig 7:**
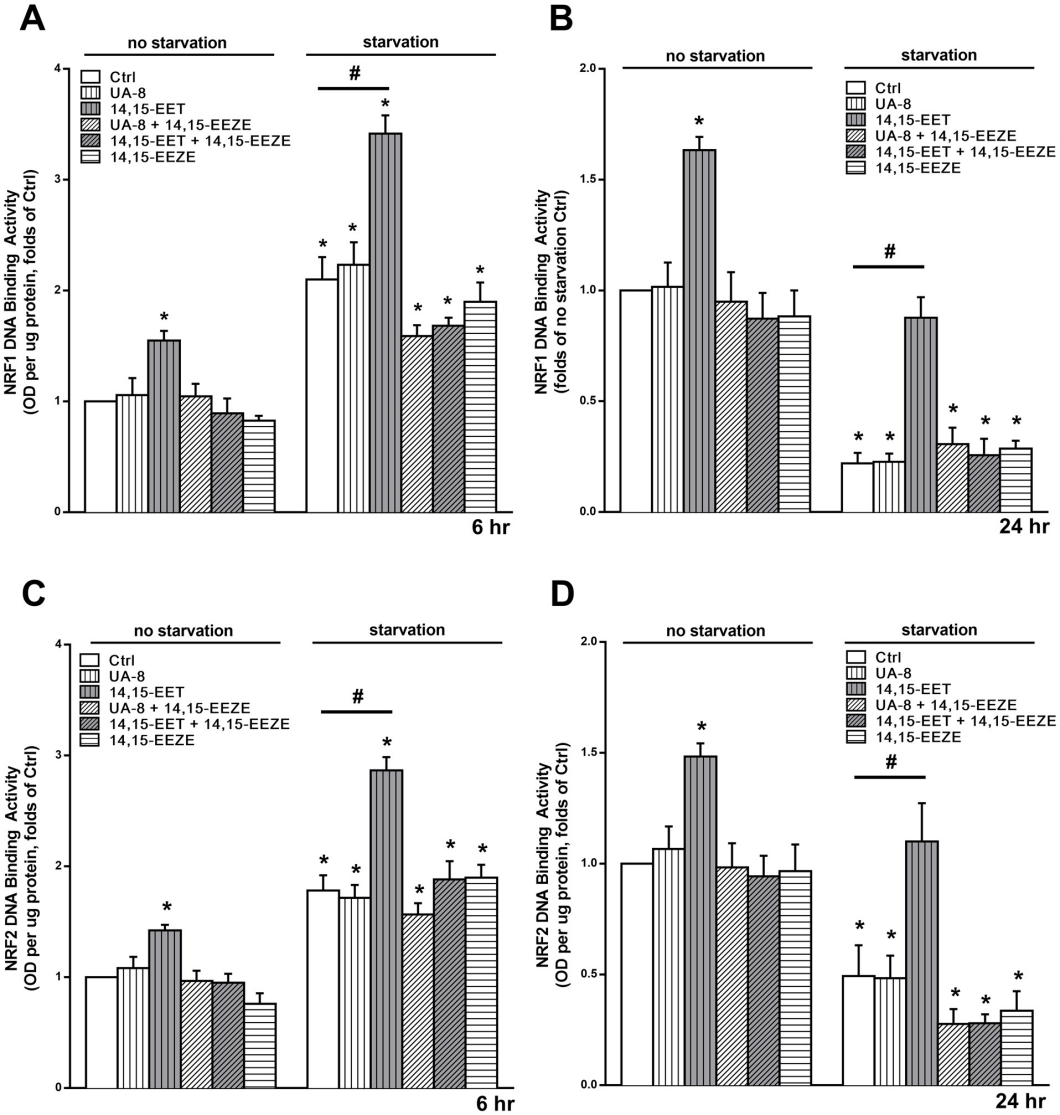
EET enhances the activity of mitochondrial biogenesis transcription factors Nrf1 and Nrf2. HL-1 cardiac cells were treated under normal cell culture conditions (NS Ctrl) or following 24h starvation (Starv) with or without UA-8 (1μM), 14,15-EET (1μM) and/or 14,15-EEZE (10μM). DNA-binding activity of Nrf1 in HL-1 cells following (**A**) 6h and (**B**) 24h and Nrf2 in HL-1 cells following (**C**) 6h and (**D**) 24h. EETs enhanced the activity of both transcription factors at all conditions and time points. Values represent mean + SEM. N≥3. *, *p<*0.05 in comparison to non-starvation control. #, *p<*0.05 between the indicated groups.

Downstream targets of PGC1 include transcription factors, nuclear respiratory factor 1 and 2 (Nrf1, and Nrf2), which promote the expression of mitochondrial respiratory complexes, mitochondrial DNA transcription factors and antioxidant defense genes [51]. We assessed the activity of Nrf1 and 2 DNA binding following 6 and 24h of starvation. Nrf1 and Nrf2 DNA binding activity was significantly increased in HL-1 cells treated with 14,15-EET, under both starvation and non-starvation conditions (Fig 7A and 7D). This effect was sustained for 24h and inhibited with the EET antagonist 14,15-EEZE. Together, these data suggest EET-mediated signaling events are involved in the regulation of mitochondrial biogenesis and energy metabolism.

## Discussion

Prolonged cellular starvation causes significant cellular damage resulting in the existence of an unhealthy pool of mitochondria. Previously, we demonstrated that HL-1 cardiac cells treated with EETs had better cell survival following 24h of starvation [31]. Thus, we are interested in understanding the mechanism(s) of EET-mediated protection, more specifically, how EETs preserve mitochondrial quality. In this study, we provide evidence that EET-mediated signaling preserves mitochondrial quality during starvation events in HL-1 cardiac cells. The EET-mediated protective response improved mitochondrial function as demonstrated by better mitochondrial respiration. Our data suggest that EETs preserve OPA1-dependent responses, which preserve mitochondrial cristae structure. In addition, we provide evidence that EET-mediated actions involve regulation of mitochondrial biogenesis leading to a better mitochondrial health, which eventually promote survival of starved HL-1 cells.

Mitochondria play a crucial role in cellular adaptation during stress by regulating the balance between cell survival and death [52]. The ability of a cell to maintain adequate ATP levels strongly correlates with the ability to avoid necrotic cell death [53]. The relative importance of mitochondria to the cell survival is highlighted by its preservation from autophagic degradation during starvation [54]. Brief starvation induces several responses, such as OPA1 oligomerization, that improve mitochondria energy production [12, 15, 23, 24]. In the current study, upregulation of mitochondrial biogenesis within the first 6h of starvation suggests the cellular strategy to invest in maintaining mitochondria quality for survival. However, prolonged starvation eventually depletes essential nutrients triggering extensive autophagic and apoptotic responses [55]. Such a switch in survival strategies was observed following 24h of starvation course as mitochondrial function and biogenesis declined. The early improvement in mitochondrial function during starvation is potentially a critical period to increase survival.

Mitochondrial quality control is a complex series of coordinated events involving turnover of damaged organelles, generation of new organelles and maintenance of function. The relative preservation of nuclear encoded mitochondrial proteins observed following 24h of starvation suggested that HL-1 cells were attempting to limit the loss of mitochondria. However, decreased expression of the mitochondrial-encoded protein COX-I at 24h indicated increased susceptibility of mitochondria to stress. Evidence has demonstrated the mitochondrial protein synthesis system is influenced by the cellular environment [56]. EET-mediated events preserved the expression mitochondrial-encoded COX-I protein, which correlated with better mitochondrial quality as confirmed by higher respiratory capacity. Citrate synthase activity has been described and well documented as a reliable marker of mitochondrial content [41]. Previously we demonstrated UA-8 had preserved citrate synthase activity in HL-1 cells subjected to 24h starvation suggesting increased mitochondrial content [31]. Moreover, as aconitase protein has been demonstrated to protect mtDNA activity [41], our data demonstrating EETs preserve aconitase activity correlates with better mitochondria. Together, these data suggest EET-mediated events preserve content and function resulting in a healthier mitochondrial pool.

Studies investigating mitochondrial response to starvation indicate a quick inhibition of mitochondrial fission shifts the balance of mitochondrial dynamics toward longer and/or branched mitochondria [15]. Excessive mitochondrial fusion or insufficient fission disrupts the balance resulting in the accumulation of compromised mitochondria over time [57]. Indeed, hyperfused mitochondria are linked to the decline in mitochondrial function seen in aging skeletal muscles [58, 59] and have been suggested to contribute to the progression of chronic diseases [13]. In the current study, EET-mediated effects limited the elongation of mitochondria. Surprisingly, while the starvation induced elongation correlated with decreased expression of DRP1 in HL-1 control cells, UA-8 treatment failed to prevent the loss of DRP-1 suggesting a different mechanism might be involved.

Our data demonstrated UA-8 treated HL-1 cells had altered OPA1 expression patterns and increased cristae density correlating with better function. OPA1 is a critical protein involved in regulating the structure of mitochondrial inner membranes into cristae thereby influencing both the integrity and functional efficiency [45, 60–62]. Metabolic stress or starvation has been shown to promote OPA1 oligomerization and increased cristae density [24]. Evidence indicates that proteolytic processing of the long form of OPA1 (L-OPA1) might be a protective stress response [23]. For example, L-OPA1 is required for mitochondrial fusion and decreased levels of L-OPA1 lower the threshold required to sense mitochondrial dysfunction resulting in better quality control [23]. Interestingly, recent reports suggest stress-induced processing of L-OPA1 led to enhanced resistance to apoptosis [23, 63]. The decreased L-OPA1 levels in the current study together with our previous data indicating EETs promote survival during starvation stress by reducing apoptosis suggesting the importance of OPA1 in the protective response [31].

Mitochondrial quality control is a complex series of coordinated events involving turnover of damaged organelles, generation of new organelles and maintenance of function. This requires coordinating mitochondrial and nuclear genomes through Nrf1/2, Tfam, SIRT1/3, CREB and PGC1*α* pathways [64]. Our current and previous data demonstrating changes to mitochondrial protein expression and increased citrate synthase activity, as a marker of increased content [31], strongly suggest EET-mediated events activate key factors required for mitobiogenesis resulting in production of new mitochondrial components. Specifically, we showed increased DNA binding activity of CREB and Nrf1/2 as well as increased enzymatic activity of SIRT1. Based on the current results, it appears both the nuclear and mitochondrial genomes are targeted by an unknown mechanism. Collectively, our data indicate that EET-mediated effects involve activation of mitobiogenesis as an inherent part of the overall protective response against starvation in HL-1 cardiac cells.

In conclusion, our new data expand on a previous study demonstrating that EET-mediated events protect HL-1 cardiac cells subjected to starvation and provide support for a hypothesis that EETs regulate mitochondrial quality [31]. Activating autophagy, specifically mitophagy, may be accompanied with concurrent induction of mitochondrial biogenesis to supplement the mitochondrial population with freshly synthesized mitochondrial proteins [65], thereby enhancing overall mitochondrial turn over to maintain higher mitochondrial quality [65]. The early adaptive response observed in control cells was lost as the stress continued but not in EET-treated cells. Thus, these data support the role of EET-mediated signaling extending the survival phase of the cellular response to starvation stress and delaying the apoptotic phase in HL-1 cardiac cells. The protective mechanisms activated by EETs involve a cascade of reactions directed to maintain a healthy pool of mitochondria promoting cell survival. Further elucidation of these mechanisms will provide insight into EET-induced cardioprotection offering new targets for therapeutic strategies.

## Supporting Information

S1 File Raw Data. (PZF)
